# A Successful Pregnancy Outcome After Continued Surveillance of Lupus Anticoagulant Antibodies in a Patient With Recurrent Pregnancy Loss: A Case Report and Literature Review

**DOI:** 10.7759/cureus.46029

**Published:** 2023-09-26

**Authors:** Hannah Flagstad, Tori E Abdalla, Yasmina Sultan, Pedro Mastrodomenico, Ellen G Wood

**Affiliations:** 1 Obstetrics and Gynecology, Midwestern University Chicago College of Osteopathic Medicine, Downers Grove, USA; 2 Medicine, Philadelphia College of Osteopathic Medicine, Philadelphia, USA; 3 Biomedical Sciences Program, Philadelphia College of Osteopathic Medicine, Philadelphia, USA; 4 Obstetrics and Gynecology, Mount Sinai Medical Center, Miami, USA; 5 Reproductive Endocrinology and Infertility, IVFMD South Florida Institute for Reproductive Medicine, Cooper City, USA

**Keywords:** low-molecular-weight heparin, lupus anticoagulant antibodies, antiphospholipid antibody, prolonged diluted russell viper venom time, autoimmunity, recurrent pregnancy loss

## Abstract

Recurrent pregnancy loss (RPL) can be a devastating experience for individuals trying to have children. Various potential causes contribute to the multifactorial pathogenesis of RPL, including chromosomal anomalies, endocrine conditions, autoimmunity, thrombophilias, and infectious agents. Antinuclear antibodies (ANAs) offer an unspecific clue to the underlying autoimmune pathogenic etiology of RPL. This case details a 40-year-old female with a history of RPL, fibromyalgia, and ANA positivity, who spontaneously developed lupus anticoagulant antibodies during her second trimester of pregnancy. Although the recommended American Society of Reproductive Medicine (ASRM) diagnostic criteria for initiating a thrombophilia evaluation was not formally met, the patient's maintenance of low-molecular-weight heparin (LMWH) throughout her pregnancy may have contributed to the success of the pregnancy. When treating a patient with RPL, consideration of the comprehensive clinical picture should precede the need to strictly adhere to published criteria for using non-proven clinical interventions. A risk-benefit analysis ought to be considered when offering patients additional medications that may come with some risks but could significantly improve the chances of a successful clinical outcome, such as live birth. We aim to provide evidence to promote greater flexibility in guidelines so that a patient's unique autoimmune etiologies of RPL are not overlooked.

## Introduction

In the United States, recurrent pregnancy loss (RPL) is described as having two or more failed clinical pregnancies [1]. The management and treatment of RPL is a complex issue with multiple possible attributing causes; however, up to 50% are idiopathic [1]. Despite RPL only occurring in 2% of pregnant patients, experiencing RPL can be devastating [1]. Several causes of RPL have been proposed, including maternal age, endocrine diseases, chromosomal anomalies, thrombophilias, autoimmune disorders, and infectious agents [2]. Of these, autoimmune disorders are believed to cause about 20% of RPL [2].

Autoimmunity is a significant risk factor to consider for evaluating potential causes of RPL. One possible indicator of an underlying autoimmune condition is the presence of antinuclear antibodies (ANAs). Even though they are not very specific, their existence may necessitate further workup. The role of a positive ANA screen, RPL, and the potential mechanism linking the two is still being hypothesized and studied [3]. However, other auto-antibodies, such as lupus anticoagulant antibodies (LACs), may be present when the ANA is positive. LACs can interfere with the clotting process because they target a component of the cell membrane, specifically the negatively charged phospholipid-protein, earning it a name as one of the antiphospholipid antibodies [4]. Testing for LAC can be essential in patients with suspected autoimmune hypercoagulable states and for the possible diagnosis of antiphospholipid syndrome (APS) [4].

Antiphospholipid syndrome, a kind of acquired thrombophilia, is an etiology of RPL due to the increased propensity to form microvascular thromboses [5]. Many treatment regimens have been suggested as a potential treatment for thrombophilias in pregnancy, but not many have been proven to yield statistically significant positive results [6,7]. This case details a 40-year-old female with a history of RPL, fibromyalgia, and ANA positivity, which developed after a failed frozen embryo transfer (FET) and then spontaneously developed LAC during her second trimester of pregnancy. Based on formal criteria put forth by the American Society of Reproductive Medicine (ASRM) 2012 Committee Opinion, the patient did not qualify for a diagnosis of antiphospholipid syndrome (APS); however, her history of autoimmune disease and prior pregnancy loss suggested the need for continued surveillance and treatment of the LAC [8].

## Case presentation

A 40-year-old woman (gravida 2, para 0) presented to the clinic seeking a comprehensive fertility evaluation. She had a history of 18 months of infertility following RPL. The product-of-conception testing for her first spontaneously conceived pregnancy showed trisomy 15, while her second spontaneously conceived pregnancy resulted in a blighted ovum and was not karyotyped. Prior to her visit, the patient had completed two previous in vitro fertilization (IVF) cycles using preimplantation genetic testing for aneuploidy (PGT-A) with another physician. The first cycle yielded 10 oocytes, seven of which were successfully fertilized. Of these, six advanced to the blastocyst stage, and trophectoderm biopsies were performed. PGT-A identified only one euploid embryo. A standard frozen embryo transfer (FET) preparation cycle was carried out, yielding a negative result.

Subsequently, the patient underwent a second IVF cycle, yielding seven retrieved oocytes, which developed into three blastocysts. PGT-A analysis identified one euploid embryo. An endometrial receptivity analysis was conducted before another FET. Her endometrium was receptive within the standard implantation window. She also tested positive for an elevated Natural Killer Cell Assay and now manifested a positive ANA screen. An expanded antiphospholipid panel was also performed, which was negative for the following: IgM/IgG cardiolipin, IgM/IgG phosphatidylethanolamine, IgM/IgG phosphatidylinositol, IgM/IgG phosphatidic acid, IgM/IgG phosphatidylserine, and IgM and IgG phosphatidylglycerol.

At presentation with a new physician, the patient expressed a desire for a third IVF attempt to create more euploid embryos. The patient's medical history included no known drug allergies. She was taking prescription Lexapro 20 mg daily and over-the-counter supplements that included coenzyme Q10 (600 mg), vitamin D (10,000 IU), pyrroloquinoline quinone (20mg), docosahexaenoic acid (DHA fish oil), dehydroepiandrosterone (DHEA 25 mg three times a day), melatonin (3 mg), and daily aspirin (81 mg). Her medical history was significant for fibromyalgia, a history of a positive ANA, and a heterozygous C677T methylenetetrahydrofolate reductase (MTHFR) mutation. Her surgical history included a hysteroscopic polypectomy. Her social history was significant for recovering from alcohol dependence, with 18 months of sobriety before initiating fertility treatment. Her family history included her mother's diagnosis of fibromyalgia but was negative for a history of thromboembolism or clotting disorders. Her 43-year-old male partner has a normal karyotype (46XY), a history of high cholesterol, and a family predisposition to heart disease. He took male fertility vitamins, fish oil, and vitamin D. His semen analysis was within normal limits. 

Preconception evaluation with her first physician encompassed a comprehensive panel for RPL and implantation failure. All results (Table 1) were negative except for a slightly elevated partial thromboplastin time-lupus anticoagulant (PTT-LA) screen (41 seconds, reference range ≤ 40), prompting consideration of potential coagulation factors playing a role in her second pregnancy loss and, possibly, her failed FET cycle. Despite negative lupus anticoagulant evaluation, an empirical decision was made to introduce Lovenox (enoxaparin) on the day of FET. She underwent another IVF cycle, yielding 14 oocytes. Four blastocysts developed and were biopsied. After PGT-A testing, two euploid blastocysts were identified. 

**Table 1:** Comprehensive RPL and Implantation Failure Panel IgG: immunoglobulin G; IgA: immunoglobulin A; IgM: immunoglobulin M.

Lab Test	Result:
Antinuclear antibody indirect immunofluorescence assay screen	Negative
Antithrombin III activity	Within normal range
Antiphospholipid antibodies	See below
Beta-2 glycoprotein IgG/IgA/IgM antibody	<9 (normal ≤ 20)
Phosphatidylserine IgG/IgA/IgM antibody	Within normal range
Cardiolipin IgG/IgA/IgM antibody	Within normal range
Chromosome analysis	XX
Factor II	Within normal range
Factor V (Leiden)	Negative
Factor VIII	81% (normal 50-180)
Factor X	112% (normal 70-150)
Factor XII	98% (normal 50-150)
Homocysteine	5.8 mmol/L (normal < 10.4)
Plasminogen activator inhibitor	6 ng/mL (normal 4-43)
Protein C	136% (normal 70-180)
Protein S	91% (normal 60-140)
Prothrombin (Factor II) 20210G > A mutation	G20210A variant not detected

Another standard FET preparation was done, and a single euploid blastocyst was transferred. Then, 8% intralipid solution was administered one week before FET due to the elevated natural killer cell assay. Then Prednisone 10 mg was started daily due to her history of a positive ANA, along with Lovenox 40 mg subcutaneously daily on the day of FET. A positive pregnancy test followed 10 days post-FET. Transvaginal sonography at an estimated six weeks and six days of gestation revealed a single intrauterine gestational sac with a yolk sac, a fetal pole of consistent crown-rump length, and a fetal heart rate of 142 beats per minute. At 12 weeks gestation, the LAC evaluation was repeated to discontinue the Lovenox at the request of the obstetrician. However, the results showed a consistently elevated PTT-LA screen at 41 seconds (Table 2). The dRVVT screen and hexagonal phase confirmation tests were consistently negative, but the decision to continue the Lovenox was made. At 16 weeks, the patient was tested again, showing an elevated PTT-LA of 95 seconds with negative confirmatory tests, and the Lovenox was continued. At 20 weeks, the patient was once again tested for the presence of lupus anticoagulant antibodies (LAC) and was found to have an elevated PTT-LA screen of 42 seconds, a dRVVT screen of 49 seconds (reference range ≤ 45), a positive dRVVT Mix interpretation, a positive dRVVT confirmation with a 1:1 mixing test that was not corrected (reference range = corrected), consistent with positive LAC. The decision was made to continue the Lovenox for the duration of the pregnancy. She was changed to subcutaneous heparin 5000 iu twice daily at 36 weeks until delivery, and continued testing was abandoned. She delivered a healthy female at 37 weeks by cesarean section due to failed induction after premature rupture of membranes.

**Table 2:** Lupus Anticoagulant Evaluation Throughout Fertility Screening, Pregnancy, and Postpartum PTT-LA: partial thromboplastin time - lupus anticoagulant; dRVVT: dilute Russell's viper venom time

Lupus Anticoagulant Results	Prior to Frozen Embryo Transfer	12 Weeks	16 Weeks Pregnant	22 Weeks	1 Year Postpartum
Lupus anticoagulant evaluation with reflex	Not detected	Not detected	Not detected	Detected	Not detected
PTT-LA screen	41 seconds (normal < 40)	41 seconds (normal < 40)	95 seconds (normal < 40)	42 seconds (normal < 40)	34 seconds (normal < 40)
dRVVT screen	40 seconds (normal < 45)	40 seconds (normal < 45)	37 seconds (normal < 45)	49 seconds (normal < 45)	32 seconds (normal < 45)
dRVVT Mix interpretation	Not indicated	Not indicated	Not indicated	Positive	Not indicated
Hexagonal phase confirm	Negative	Negative	Negative	Negative	Not indicated
dRVVT confirm	Not indicated	Not indicated	Not indicated	Positive	Not indicated
dRVVT 1:1 mix	Not indicated	Not indicated	Not indicated	Not corrected (normal = corrected)	Not indicated

She subsequently returned to the infertility clinic to attempt another FET with one of the two of her remaining euploid embryos. She was later retested for LAC, yielding a negative result; however, her ANA remained positive at 1:40 titer with a nuclear, speckled pattern. The same FET protocol yielded a negative result with her first euploid embryo and a positive result with her second euploid embryo. Her second FET after her successful pregnancy unfortunately ended in a blighted ovum. 

## Discussion

While RPL has been defined and managed differently over the years, criteria and recommendations have been debated, especially when the cause of recurrent loss is unclear. This case presents a scenario of RPL that was evaluated and managed differently than the criteria set forth by the governing guidelines suggested. In 2011, the Royal College of Obstetricians and Gynaecologists (RCOG) defined RPL as the loss of three or more pregnancies, and the American Society for Reproductive Medicine (ASRM) 2012 guidelines adjusted the definition as having two consecutive losses of a clinical pregnancy defined by ultrasound or histological examination [8,9]. In 2017, the European Society of Human Reproduction (ESHRE) described RPL as having two consecutive clinical pregnancy losses. However, they took a step back, not defining whether the losses must be consecutive [9]. While this patient's case did not meet the RCOG definition of RPL, she did meet the criteria defined by the ASRM and ESHRE, indicating that further workup to determine the etiology of her RPL should be completed. 

As part of this patient's assessment, she was evaluated extensively for various causes of RPL, including a general medical history and a workup for genetic factors, hereditary thrombophilia, APS, endocrine causes, uterine anomalies, male factor, and immunology. The only pertinent positives associated with the workup were a positive ANA consistent with prior testing and a mild positive PTT-LA screen of 41 (normal < 40 seconds) that resulted in negative confirmation testing for lupus anticoagulant on the Evaluation with Reflex test (Table 2). Lupus anticoagulants are a group of antibodies tested for in the evaluation of APS, and they encompass a diverse group of immunoglobulins that selectively focus on the epitopes found within cell membrane-associated negatively charged phospholipid-binding protein, prothrombin, and beta2-glycoprotein I (beta2-GPI). These antibodies effectively impede phospholipid-dependent coagulation when examined in vitro [10]. Criteria for diagnosing APS are defined by the 2006 Sydney revision of the original 1999 Sapporo criteria described as the persistent presence of IgG/IgM anticardiolipin antibodies (aCL), anti-B2-glycoprotein I antibodies (Anti-B2-GPI) or LAC on at least two separate occasions, 12 weeks apart, while also including clinical criteria of vascular thrombosis or pregnancy morbidity [11]. However, the dynamic nature of these antibodies can pose confusion regarding diagnosis and treatment decisions. Due to the complicated nature of screening for APS throughout pregnancy, some researchers have suggested that pregnancy may trigger an underlying APS and recommended that in patients with unexplained miscarriage, screening for APS both before and throughout pregnancy may be advisable [12]. 

To better understand this patient's initial negative evaluation, it is imperative to know how laboratories test for these antibodies despite a positive screening result. A systemic approach to diagnosing LAC entails three crucial phases: screening tests, mixing studies, and confirmatory tests [4]. Screening tests initially identify a potential LAC presence by detecting prolonged clotting times in one of two coagulation tests that are phospholipids dependent, such as PTT-LA or dRVVT [10]. Mixing studies then evaluate the influence of LAC on coagulation by blending the patient's plasma with normal plasma with the assumption that the normal plasma will correct any clotting abnormalities. At the same time, LAC interferes with the coagulation process and results in prolonged clotting times even after mixing [4]. Confirmatory tests validate the presence of LAC using dilute Russell viper venom time (dRVVT) or hexagonal phospholipid neutralization assay (HEP) [13]. dRVVT measures the prolongations of clotting times induced by diluted Russell viper venom, while HEP employs hexagonal-phase phospholipid to neutralize the effects of LAC [4]. Both of these tests help to provide a definitive diagnosis for LAC. 

The complex nature of antibodies and the varying epitopes on proteins within phospholipids make the laboratory identification of LAC demanding. As a result of this complexity, a single test for LAC cannot encompass all its variations. Clinical laboratories exhibit discrepancies in terms of sensitivity and specificity when performing these tests. Various factors contribute to this variability, including the adequacy of plasma preparation, avoidance of platelet contamination, and the dilutional impacts stemming from mixing studies, all of which influence the accurate detection of LAC [4]. Furthermore, several mechanisms are suggested to induce antiphospholipid binding proteins, such as infections, oxidative stress, and significant stress, such as surgery or trauma [14]. Due to the variety of possible triggers and the dynamic nature of these antibodies, it is important to consider further evaluation in those with positive screening tests and a history of RPL.

The decision to empirically treat the patient with low-molecular-weight heparin (LMWH) was made after a comprehensive clinical analysis that included her history of recurrent loss, a persistently prolonged LAC screening test, and her positive ANA that may suggest an underlying autoimmune process. Both the presence of autoimmune diseases, such as systemic lupus erythematosus, rheumatoid arthritis, autoimmune thrombocytopenia, and autoimmune hemolytic anemia, and the presence of pregnancy complications are indications to search for LAC in patients with a partial thromboplastin time-lupus anticoagulant (PTT-LA) [4,15]. While PTT and PTT-LA assess clotting times in patients suspected of abnormal clotting function, PTT-LA specifically assesses the intrinsic pathway with increased sensitivity to interference caused by LAC [16]. Therefore, a negative PTT does not necessarily mean that LAC is absent and may require a follow-up PTT-LA to assess for LAC. Following initiation of LMWH on the day of FET, the patient was followed closely with repeat LAC testing every four weeks, which resulted in a positive dRVVT, confirming the presence of LAC. Continued monitoring of the patient's PTT-LA during pregnancy, despite potential prolonged results from treatment with LMWH, posed a low risk and increased clinical benefit throughout pregnancy, given the patient's history of RPL, autoimmune conditions, and use of assisted reproductive technology to achieve pregnancy [17]. 

While it is impossible to know whether the pregnancy would have resulted in live birth if the patient were evaluated one-dimensionally, only considering the initially negative hexagonal phase confirmation testing, it is vital to consider the risks and benefits of treating with LMWH. The effect of LMWH in treating women with recurrent miscarriages who tested negative for antiphospholipid antibodies was evaluated. It found a significant increase in women who continued their pregnancy beyond 20 weeks compared to the control group [18]. In a systematic review and meta-analysis of LMWH and RPL, it was concluded that LMWH therapy may increase live birth rates and decrease miscarriage rates in patients with RPL in comparison to those in control groups [19]. When comparing enoxaparin, a type of LMWH, to aspirin in pregnancy, enoxaparin was associated with a significantly lower occurrence of pregnancy loss and similar bleeding rates [20]. These findings suggest that LMWH can play a role in patients with a history of RPL despite this patient not meeting all the criteria for APS. It is essential to evaluate the patient's entire clinical picture, including their risk of bleeding and history of heparin-induced thrombocytopenia when deciding how to treat a patient with LAC. However, the dynamic nature of antiphospholipid antibodies, varying specificity and sensitivity in laboratory testing, and low-risk nature of empirically dosed LMWH in patients with RPL compared to aspirin alone suggest the need to re-evaluate treatment criteria in these patients.

## Conclusions

This case offers valuable insights into the complexities surrounding the presence of LAC in diagnosis and treatment in the context of pregnancy, particularly in individuals with RPL. These antibodies' dynamic nature and impact on coagulation can complicate diagnosis and therapeutic decision-making. The presented approach to diagnosis, involving screening tests, mixing studies, and confirmatory tests, underscores the multifaceted nature of identifying LAC accurately.

The benefit of utilizing PTT-LA as a proxy for acquired APS in a patient with RPL, despite initially negative lupus anticoagulant evaluation with reflex results and a positive ANA test, cannot be overstated. In cases where standard diagnostic tests do not explicitly confirm APS, PTT-LA can offer valuable insights. Such was the case for our patient, who had experienced RPL and tested positive for ANA, raising suspicion of an underlying autoimmune condition. Although the lupus anticoagulant evaluation initially yielded negative results, continued monitoring with PTT-LA proved instrumental. At 22 weeks into the pregnancy, the lupus anticoagulant evaluation with a reflex test finally turned positive, prompting the continuation of appropriate treatment until the end of the pregnancy. This success story underscores the importance of comprehensive and vigilant diagnostic approaches, which can ultimately lead to life-changing outcomes for patients with APS.

The diagnostic journey outlined in this case exemplifies the challenges in managing LAC presence during pregnancy. The dynamic interplay between these antibodies and the coagulation system underscores the need for a comprehensive diagnostic approach considering laboratory results and clinical presentation. Moreover, the case underscores the importance of vigilance in monitoring the presence of LAC in ANA-positive patients throughout pregnancy to ensure the well-being of both the mother and the developing fetus.

In conclusion, this case study highlights the intricate diagnostic and therapeutic considerations involved in managing LAC presence in the context of RPL and infertility. It serves as a reminder of the complexity of immune-mediated factors in reproductive health and the importance of a tailored approach to diagnosis and treatment. By shedding light on the challenges encountered and decisions made throughout the diagnostic process, this case contributes to the broader understanding of LAC's impact on pregnancy outcomes. It emphasizes the need for continued research and clinical vigilance in this area.

## References

[1] Pillarisetty LS, Mahdy H (2023). Recurrent pregnancy loss. StatPearls [Internet].

[2] Turesheva A, Aimagambetova G, Ukybassova T (2023). Recurrent pregnancy loss etiology, risk factors, diagnosis, and management. Fresh look into a full box. J Clin Med.

[3] Liu T, Guo X, Liao Y, Liu Y, Zhu Y, Chen X (2022). Correlation between the presence of antinuclear antibodies and recurrent pregnancy loss: a mini review. Front Endocrinol (Lausanne).

[4] Rasool ZS, Tiwari V (2022). Biochemistry, lupus anticoagulant. StatPearls [Internet].

[5] Alecsandru D, Klimczak AM, Garcia Velasco JA, Pirtea P, Franasiak JM (2021). Immunologic causes and thrombophilia in recurrent pregnancy loss. Fertil Steril.

[6] Hamulyák EN, Scheres LJ, Marijnen MC, Goddijn M, Middeldorp S (2020). Aspirin or heparin or both for improving pregnancy outcomes in women with persistent antiphospholipid antibodies and recurrent pregnancy loss. Cochrane Database Syst Rev.

[7] Alalaf S (2012). Bemiparin versus low dose aspirin for management of recurrent early pregnancy losses due to antiphospholipd antibody syndrome. Arch Gynecol Obstet.

[8] (2012). Evaluation and treatment of recurrent pregnancy loss: a committee opinion. Fertil Steril.

[9] Chester MR, Tirlapur A, Jayaprakasan K (2022). Current management of recurrent pregnancy loss. Obstet Gynecol.

[10] Tripodi A (2007). Laboratory testing for lupus anticoagulants: a review of issues affecting results. Clin Chem.

[11] Zohoury N, Bertolaccini ML, Rodriguez-Garcia JL (2017). Closing the serological gap in the antiphospholipid syndrome: the value of “non-criteria” antiphospholipid antibodies. J Rheumatol.

[12] Honig A, Engel JB, Segerer SE, Kranke P, Häusler S, Würfel W (2010). Pregnancy-triggered antiphospholipid syndrome in a patient with multiple late miscarriages. Hum Reprod.

[13] Pengo V, Bison E, Banzato A, Zoppellaro G, Jose SP, Denas G (2017). Lupus anticoagulant testing: diluted Russell viper venom time (dRVVT). Methods Mol Biol.

[14] Passam FH, Rahgozar S, Qi M (2010). Beta 2 glycoprotein I is a substrate of thiol oxidoreductases. Blood.

[15] Miyakis S, Lockshin MD, Atsumi T (2006). International consensus statement on an update of the classification criteria for definite antiphospholipid syndrome (APS). J Thromb Haemost.

[16] Simmons DP, Herskovits AZ, Battinelli EM, Schur PH, Lemire SJ, Dorfman DM (2018). Lupus anticoagulant testing using two parallel methods detects additional cases and predicts persistent positivity. Clin Chem Lab Med.

[17] De Kesel PM, Devreese KM (2020). The effect of unfractionated heparin, enoxaparin, and danaparoid on lupus anticoagulant testing: can activated carbon eliminate false-positive results?. Res Pract Thromb Haemost.

[18] Shaaban OM, Abbas AM, Zahran KM, Fathalla MM, Anan MA, Salman SA (2017). Low-molecular-weight heparin for the treatment of unexplained recurrent miscarriage with negative antiphospholipid antibodies: a randomized controlled trial. Clin Appl Thromb Hemost.

[19] Jiang F, Hu X, Jiang K, Pi H, He Q, Chen X (2021). The role of low molecular weight heparin on recurrent pregnancy loss: a systematic review and meta-analysis. Taiwan J Obstet Gynecol.

[20] Jacobson B, Rambiritch V, Paek D, Sayre T, Naidoo P, Shan J, Leisegang R (2020). Safety and efficacy of enoxaparin in pregnancy: a systematic review and meta-analysis. Adv Ther.

